# Racial and Ethnic Differences in Traumatic Brain Injury Outcomes From 2009 to 2023: Protocol for a Systematic Review

**DOI:** 10.2196/58763

**Published:** 2024-10-03

**Authors:** Monique R Pappadis, Kelli G Talley, Patricia Garcia, Caitlin R Aguirre, Chinedu K Onwudebe, Michelle Smith, Anthony H Lequerica

**Affiliations:** 1 Sealy Center on Aging The University of Texas Medical Branch Galveston, TX United States; 2 Department of Population Health and Health Disparities School of Public and Population Health The University of Texas Medical Branch Galveston, TX United States; 3 Brain Injury Research Center TIRR Memorial Hermann Houston, TX United States; 4 Department of Rehabilitation Counseling College of Health Professions Virginia Commonwealth University Richmond, VA United States; 5 Department of Physical Medicine and Rehabilitation, TBI Model Systems School of Medicine Virginia Commonwealth University Richmond, VA United States; 6 Department of Neurology Indiana University School of Medicine Indianapolis, IN United States; 7 Department of Physical Medicine and Rehabilitation Indiana University School of Medicine Indianapolis, IN United States; 8 School of Public and Population Health The University of Texas Medical Branch Galveston, TX United States; 9 John Sealy School of Medicine The University of Texas Medical Branch Galveston, TX United States; 10 NYU Grossman School of Medicine New York University New York, NY United States; 11 Department of Research Rusk Rehabilitation New York, NY United States; 12 Kessler Foundation East Hanover, NJ United States; 13 Department of Physical Medicine and Rehabilitation Rutgers-New Jersey Medical School Newark, NJ United States

**Keywords:** traumatic brain injury, TBI, health disparities, racial and ethnic differences, injury outcomes, outcomes assessment, social determinants of health, neurorehabilitation, evidence-based interventions

## Abstract

**Background:**

In 2009, Gary and colleagues reviewed prior research examining racial and ethnic differences in outcomes after traumatic brain injury (TBI). Over the past 15 years, advances in research and changes in the demographic composition of the United States warrant a comprehensive understanding of racial and ethnic disparities after TBI.

**Objective:**

A systematic review will be conducted to examine racial and ethnic differences in TBI outcomes from 2009 to 2023.

**Methods:**

Preliminary searches and study screening processes will identify relevant English-language articles published from January 2009 to December 2023 using the CINAHL, Gale OneFile, PsycINFO (Ovid), and PubMed electronic databases. Relevant articles will include quantitative or mixed method approaches, involve individuals with TBI or their caregivers, and compare 2 or more groups by race or ethnicity on post-TBI outcomes. Quality will be assessed using the Newcastle-Ottawa Scale. This systematic review protocol was developed following PRISMA-P (Preferred Reporting Items for Systematic Review and Meta-Analysis Protocols) guidelines. Results will be summarized, and a subgroup analysis may be conducted based on demographics (eg, age, gender, or sex).

**Results:**

We have already identified abstracts using the search strategy for all 4 of the included electronic databases. We recently updated the search and will begin abstract screening of the additional abstracts identified from the last search completed in January 2024. This systematic review is anticipated to be completed by fall 2024, and its findings will be disseminated to the scientific community, persons with TBI, caregivers, and the lay audience.

**Conclusions:**

This systematic review will advance our understanding regarding outcome disparities among minoritized individuals with TBI, examine progress over the past 15 years in minimizing barriers encountered by these racial and ethnic groups, and provide professionals with a roadmap illustrating existing gaps in rehabilitation care, making way for further development and implementation of evidence-based interventions to improve health equity in TBI outcomes.

**Trial Registration:**

PROSPERO CRD42023394529; https://tinyurl.com/53mtcz9b

**International Registered Report Identifier (IRRID):**

PRR1-10.2196/58763

## Introduction

The estimated global prevalence of traumatic brain injury (TBI) is over 55 million, a number that has continued to grow over the past 3 decades [1]. From 1990 to 2016, the incidence of TBI increased by 3.6% and prevalence increased by 8.4%; in 2016 alone, there were 27 million new cases [1]. From these estimates, approximately 0.7% of the world’s population live with a medically treated brain injury. TBI is prevalent among all age groups and commonly causes long-term functional, cognitive, behavioral, and social challenges that require a multidisciplinary approach to rehabilitation [2,3]. As emphasized by the 2022 US National Academies of Sciences, Engineering, and Medicine report [4], one’s ability to access appropriate care and maximize functional recovery is dependent upon numerous personal and social factors. These factors include race, ethnicity, age, gender, socioeconomic status, geographical location, preinjury status, and the presence of comorbidities, among others [5]. Of these social determinants of health (SDoH), racial and ethnic disparities are the most reported health disparities following TBI [6].

Earlier studies of racial and ethnic health disparities following TBI rehabilitation using large databases focused primarily on adults categorized as “minorities versus Whites.” The seminal paper written by the early pioneer in the TBI field examined the impact of *minority status* on functional outcomes using the TBI Models Systems national database, which included 586 African American (37%), Hispanic (7%), and White (53%) individuals categorized as “minority versus Whites” [7]. Rosenthal et al [7] used the Community Integration Questionnaire and determined that minority status affected long-term community integration (CI), with adults with TBI from racial and ethnic minority groups faring worse in long-term productivity and social integration compared to White adults. With time, the TBI field began to conduct more rehabilitation research focused mainly on adults.

In the early and mid-2000s, TBI researchers began centering on race as a moderator of functional outcomes following TBI [8-10]. By the mid- to late 2000s, research on racial and ethnic minority status and TBI exploded, and numerous studies narrowed around postinjury outcomes such as CI, competitive employment, job stability, and life satisfaction. In these studies, “minority,” “Hispanic,” or “Black” adults reported worse outcomes in regard to functioning, employment, life satisfaction, and CI relative to White adults with TBI [11-17] and without significant differences between minority groups [11], even after accounting for income [17].

When exploring outcomes after TBI, the needs of caregivers of persons with TBI are important factors that must be addressed. Racial differences in kinship patterns have been identified, with African American caregivers spending significantly more time in direct caregiving and reporting more depression than White caregivers [18]. Black and Hispanic caregivers made greater use of emotion-focused coping skills and used more traditional ideology with a greater sense of obligation and family duty. Interestingly, caregiving did not lead to greater perceived burden [19]. By 2009, there were no studies specifically examining racial and ethnic differences among older adults with TBI. However, within the past decade, more studies have examined disparities in care and outcomes among older adults with TBI.

In more recent years, interest has increased in understanding how racial and ethnic minority groups are differentially impacted by a TBI. While TBI sequelae largely depends on specific aspects of the brain injury itself and patient characteristics (eg, age, premorbid medical health, and psychiatric health), the effects from the brain injury occur in a social and cultural context. Recent systematic reviews have tried to shed light on racial and ethnic disparities in TBI outcomes. Their findings align with many of the conclusions by Gary et al [14], suggesting that racial and ethnic disparities exist across acute care, rehabilitation, and long-term outcomes and in domains of community reintegration, degree of disability, and return to work when compared to non-Hispanic White individuals [20]. Race and ethnicity are also the most prevalent SDoH implicated in the incidence of TBI [6]. We recognize that both interpersonal and structural racism are often the drivers of disparities experienced by minoritized racial and ethnic groups [21].

Prior reviews have either been non-TBI specific, included other clinical groups such as stroke and cardiac arrest [22], excluded patients with mild TBI [23], focused exclusively on health utilization during acute care [22], or included studies that had narrow outcomes such as mechanisms of injury and other pathophysiology in racial and ethnic minority groups [24]. Although not a systematic review, the work by Gary et al [14] inspired our proposed work. Therefore, we aim to conduct a rigorous comprehensive analysis of the literature over a 15-year period focused on racial and ethnic disparities in TBI outcomes (eg, treatment, functional, psychosocial, emotional or behavioral, and caregiver) across the lifespan and including all injury severities. We hypothesized that racial and ethnic disparities in outcomes persist across all outcome domains and across the continuum of care regardless of age or injury severity.

## Methods

### Study Design

Following the PRISMA-P (Preferred Reporting Items for Systematic Review and Meta-Analysis Protocols) guidelines (Multimedia Appendix 1) [25], we outline an updated systematic review of quantitative and mixed methods studies reporting racial and/or ethnic differences in TBI outcomes across the lifespan. The systematic review protocol is registered in PROSPERO (CRD42023394529).

### Eligibility Criteria

The PICOS (Patient, Intervention, Comparison, Outcomes, and Study Design) framework was used to develop our systematic review question and guide the development of the literature search strategies [26]. The elements are detailed below:

- Participants (P): Individuals with a TBI of any age and any severity. Studies including people with an acquired brain injury (eg, stroke, tumor, and hypoxic injury) in addition to TBI may be included if the results for persons with TBI are separated. If there are sufficient studies using pediatric, adult (age >18 years), or older adult (aged 55+ years) populations or caregivers, then we will decide if we want to separate the findings and develop separate review articles for each population.
- Intervention (I): Not applicable.
- Comparison (C): Racial or ethnic group identity, where 2 or more ethnic or racial groups must be compared. Comparisons between “majority” and “minority group” and between “non-Hispanic White” and “ethnic or racial minority group” are examples of other comparisons that may be included. We will acknowledge the earlier race or ethnicity language used in earlier studies, but our review will use current, inclusive race or ethnicity language.
- Outcomes (O): Outcomes following TBI, such as treatment, function, psychosocial, cognition or neuropsychological, and emotional or neurobehavioral. Studies may also address caregiver outcomes.
- Study design (S): All quantitative or mixed methods studies (eg, observational research, cohort study, prospective, and longitudinal) will be included. Qualitative studies, abstracts, editorials, review articles, commentaries, dissertations or theses, case reports, policy statements, and epidemiological studies will be excluded. All included papers will be in the English language and published from January 1, 2009, to December 31, 2023.

The Determinants of Inequities in Neurological Disease, Health, and Well-being framework by the National Institute of Neurological Disorders and Stroke will be used to also identify the individual-to-structural determinants of health reported in the included studies [21]

### Information Sources

We have performed preliminary searches and piloted the study screening process using the CINAHL, Gale OneFile, PsycINFO (Ovid), and PubMed electronic databases to identify relevant quantitative or mixed method articles published in English. On January 9, 2023, we conducted a preliminary database search to ensure the inclusion of literature from January 1, 2009, to December 31, 2022. We recently conducted our final search on January 10, 2024, to identify relevant articles published since the last search through December 2023. We will hand search the reference lists of relevant systematic reviews and articles to identify additional studies not previously identified. All references identified through the searches will be exported into the online review software Covidence. Duplicates are removed automatically by the Covidence software.

### Search Strategy

A variety of Medical Subject Headings and relevant key words were developed and used by a medical librarian with significant experience in performing literature searches. The full search strategies for each database are included in Multimedia Appendix 2. Results were filtered by dates and English language.

### Study Selection

Using Covidence, at least 2 of the 7 reviewers will independently screen each reference according to the title and abstract. All disagreements will be resolved either by the pair or by the inclusion of a third reviewer. During the initial preliminary search, we were able to identify 1746 records that were imported for screening (693 from CINAHL, 507 from PubMed, 326 from PsycINFO, and 220 from Gale OneFile). Of these, 520 duplicate references were automatically removed by Covidence. An additional 326 records were identified and added to Covidence (61 from CINAHL, 200 from PubMed, 21 from PsycINFO, and 44 from Gale OneFile) during the updated search through December 2023, and 103 duplicates were removed. We updated our search criteria to include Alaska Native, resulting in an additional 47 records identified (11 from CINAHL, 22 from PubMed, 10 from PsycINFO, and 4 from Gale OneFile). As a result, we added 12 additional abstracts after 35 duplicates were removed. We will begin the review of the 235 additional abstracts included. Upon abstract review completion and resolving any disagreements, we will begin the full-text screening phase. The full-text articles will be uploaded to Covidence to facilitate the review. Each full-text reference will be reviewed for inclusion by 2 independent reviewers out of the 7 total reviewers. Any disagreements will be resolved as a group. Full-text articles will be included in the data abstraction or collection phase. We will hand search the reference lists of relevant review articles that were flagged during the abstract and full-text screening phase, as well as review the references of the included full-text articles. We will also use hand searching methods to manually identify any relevant studies not identified through our included databases. A PRISMA (Preferred Reporting Items for Systematic Review and Meta-Analysis) flow diagram will document the selection process, including the reasons for exclusions.

### Data Collection

The review team will meet to reflect on the full-text review process and included articles, then finalize the data extraction form and its elements for inclusion in Covidence. We will pilot test the data extraction form on at least 4 of the included articles before completing the process with the remaining included full-text articles. We will follow the same review process as before by having the reviewers work independently in pairs to extract data for each included article within Covidence. A third reviewer will resolve any discrepancies. If needed, the group will discuss the discrepancies to finalize the included data. If data are not clear in the article, the investigators will contact the authors to confirm.

### Data Items

The following data will be extracted from each of the included articles: source reference; publication year; population (ie, pediatric populations, adults aged ≥18 years, or older adults aged ≥55 years); race or ethnicity; sex or gender; geographic setting (eg, within or outside of the United States); study setting (eg, acute or inpatient, rehabilitation, outpatient, or community); sample characteristics; sample size; nature of study (descriptive, quantitative, or mixed methods); study design (eg, cross-sectional, cohort, or longitudinal); description and type of individual-, interpersonal-, institutional-, community-, or structural-level factors discussed (eg, socioeconomic status, health care coverage, housing, citizenship status, neighborhood location, or food security); measures of effect (eg, proportions, relative risks, odds ratios, or hazard ratios); outcome measures; and summary of author interpretations or conclusions.

### Risk of Bias and Quality Assessment

Two investigators out of the 7 will independently assess the methodological quality of each individual article based on the Newcastle-Ottawa Quality Assessment Scale [27]. A third reviewer will address any disagreements, or the team will meet to discuss and decide as a group. If needed, study authors will be contacted to provide additional details or clarification.

### Outcomes

Primary post-TBI outcomes include treatment outcomes (eg, emergency department, discharge disposition, rehabilitation placement or access, and rehabilitation treatment), mortality, functional outcomes (eg, functional status at admission, discharge, and 1+ years follow-up using the Functional Independence Measure, Disability Rating Scale, and Functional Status Examination instruments), psychosocial outcomes (eg, employment or productivity, community integration, marital status, quality of life or life satisfaction, and driving), cognitive or neuropsychological outcomes (eg, executive functioning, processing speed, and working memory), and emotional or neurobehavioral outcomes (eg, posttraumatic stress, postconcussion disorder, depression, anxiety, and aggression). Studies may also address as secondary outcomes those of caregivers of people with TBI (eg, caregiver burden, family functioning, psychological symptoms, posttraumatic stress disorder, and quality of life). However, if sufficient studies are identified based on caregiver outcomes, then we will report the findings in a separate review.

### Data Synthesis

We plan to conduct a narrative synthesis instead of a meta-analysis due to the high likelihood of heterogeneity and the diversity of confounding variables and the data included in the studies. For the narrative synthesis, we will synthesize the types of studies, methodologies used, methodological considerations, findings of the studies (particularly regarding racial and ethnic differences in outcomes), evidence of findings, limitations of methods, and a summary of author conclusions. If sufficient data are identified, we will conduct a subgroup analysis by age (eg, older adults or pediatric populations) or by sex or gender.

### Ethical Considerations

No ethical approval from an institutional review board was required for this systematic review.

## Results

As mentioned previously, the initial protocol has been registered in PROSPERO (CRD42023394529). We have already identified abstracts using the search strategy for all 4 of the included electronic databases. We recently updated the search and will begin abstract screening of the additional abstracts identified from the last search completed in January 2024. We will then begin full-text review, and we anticipate that entire systematic review will be completed by fall 2024. The study results will be disseminated through an open-access publication in a peer-reviewed journal, and we will present our findings at an international or national meeting. We also hope to develop a nontraditional dissemination product that can be shared with persons with TBI, their families, and the lay audience.

## Discussion

### Expected Findings

The goal of this work is to seek and review additional literature regarding outcomes of minoritized populations with TBI across the recovery process. Outcomes of interest are broad and inclusive of physical and cognitive functioning, as well as emotional and behavioral outcomes. This work will also review treatment outcomes in studies examining differences between minoritized and nonminoritized communities. This comprehensive review builds upon and updates decades-long research examining racial and ethnic disparities in TBI outcomes as the field continues to investigate ways to achieve health equity in the population with TBI.

This work is especially appropriate currently as many fields of research increase focus on SDoH [21,28], which affect all areas of a person’s environment, from education to health care access and quality, to economic stability and the social context. With the increased awareness and inclusion of SDoH in research since 2009, this review can contribute to the identification of higher-level intervention opportunities in terms of clinical and community programming, as well as policy changes affecting treatment across numerous points in the continuum of care. With this review, we expect to gain additional insight into progress or continuing barriers to health equity and be able to identify any gaps in the literature in the science of treatment and recovery for minoritized populations with TBI.

### Study Limitations

Limitations of our study include the use of only English-language studies, which can limit the representativeness of the review. Our study is also limited by the validity of the articles we include. The heterogeneity of the articles can make it difficult to draw conclusions; however, to facilitate interpretation, we will summarize the findings based on outcomes of interest. The definitions of race and ethnicity are informed socially and are continuously changing; these definitions could change in the future and alter the implications of our review. The treatment of racial and ethnic groups as homogenous could also lead to erroneous conclusions. We focused only on racial and ethnic social identities; therefore, we must acknowledge that there are multiple dimensions of social identities that may influence disparities in outcomes after TBI.

### Conclusions

The results of this updated systematic review could provide greater insights into ethnic and racial disparities experienced following TBI across the lifespan. We hope that the results of this updated systematic review will not only corroborate the findings of Gary et al [14] and others, but also add to the knowledge base regarding post-TBI outcome differences by race or ethnicity. It will also include a comprehensive analysis of disparities across the various stages of recovery, outcomes, and settings and will use rigorous and systematic processes of review, data collection, and extraction. Instead of being one of many disparities-focused reviews, we hope that our findings will be serve as foundational knowledge to inform clinical practice; guide the development of patient-centered and culturally-guided services, programs, and interventions to address identified disparities; as well as to inform policy and practice changes to address systemic inequities that continue to perpetuate health disparities for marginalized communities with TBI.

## Appendix

Multimedia Appendix 1 Search strategies for the CINAHL, Gale OneFile, PsycINFO (Ovid), and PubMed databases.

Multimedia Appendix 2 PRISMA-P (Preferred Reporting Items for Systematic Review and Meta-Analysis Protocols) 2015 checklist.

## Abbreviations

CI: community integration
PICOS: Patient, Intervention, Comparison, Outcomes, and Study Design
PRISMA: Preferred Reporting Items for Systematic Review and Meta-Analysis
PRISMA-P: Preferred Reporting Items for Systematic Review and Meta-Analysis Protocols
SDoH: social determinants of health
TBI: traumatic brain injury
