# Supplementary material for: Racial and Ethnic Differences in Traumatic Brain Injury Outcomes From 2009 to 2023: Protocol for a Systematic Review
Source: JMIR Res Protoc. 2024 Oct 3;13:e58763. doi: 10.2196/58763 (PMC11487201; doi:10.2196/58763)
Supplement: Multimedia Appendix 2 [file resprot_v13i1e58763_app2.pdf]

This is a Multimedia Appendix to a full manuscript published in the J Med Internet Res. For full copyright and citation information see http://dx.doi.org/10.2196/jmir.58763.

Database	Search Terms
CINAHL(EBSCO)	1. (MH "Brain Injuries+") OR "traumatic brain injur*" OR "brain injur*" OR "head injur*"<br>2. (MH "Brain Concussion+") OR (MH "Postconcussion Syndrome") OR concuss*<br>3. S1 OR S2<br>4. (MH "Treatment Outcomes+") OR (MH "Outcome Assessment") OR (MH "Patient-Reported Outcomes+") OR "functional outcome*" OR outcome*<br>5. (MH "Minority Groups") OR (MH "Healthcare Disparities") OR "minority health"<br>6. ((cultural or racial) N3 difference*)<br>7. (MH "Ethnic Groups+") OR (MH "Race Factors") OR ("race factor*" or "ethnic group*" or race* or racial or "racial disparit*" or "racial minorit*" or ethnicit* or "ethnic disparit*" or "ethnic minorit*" or ethnic* or minorit* or culture*)<br>8. (MH "Black Persons") OR (negro* or black* or "african american*" OR "afro american*" or afroamerican*)<br>9. (MH "Hispanic Americans") OR ("spanish american*" or "Puerto Rican*" or latina* or Cuban* or hispanic* or latino* or "mexican american*")<br>10. (MH "Native Americans+") OR ("native american*" or "american indian*" OR indigenous*)<br>11. (MH "Asians+") OR (asian* or "pacific islander*")<br>12. (MH "Alaska Natives+") OR (MH "Inuit+") OR ("american indian*" or "american native*" or "alaska* native*" or "alaska* indigenous people*" or "indigenous alaska*" or "native

	alaska*" or inuit* or eskimo* or inuk* or aleut* or kalaallit* or inupiat* or alaska*)<br>13. S5 OR S6 OR S7 OR S8 OR S9 OR S10 OR S11 OR S12<br>14. S3 AND S4 AND S13 – Limiters: - Publication Date: 20090101-20240131; English Language<br>15.
Gale OneFile	Keyword: ("traumatic brain injury" or "brain injury" or "head injury" or concussion) AND Keyword: ("functional outcome" or outcome) AND Keyword: (race or ethnicity or minorit* or culture or "minority health" or black* or "african american*" or "spanish american*" or latin* or hispanic or "mexican american" or "american indian*" or asian* or indigenous* or alaska*)
PsycINFO(Ovid)	1. exp Traumatic Brain Injury/ or "traumatic brain injur*".ti,ab. or tbi.ti,ab.<br>2. exp Brain Injuries/ or "brain injur*".ti,ab. or "head injur*".ti,ab.<br>3. exp Brain Concussion/ or concuss*.ti,ab.<br>4. 1 or 2 or 3<br>5. exp Treatment Outcomes/ or exp "Treatment Process and Outcome Measures"/ or exp Cross Cultural Treatment/ or exp Patient Reported Outcome Measures/ or "functional outcome*".ti,ab. or outcome*.ti,ab.<br>6. exp Minority Groups/ or exp Health Disparities/ or "minority health".ti,ab.<br>7. exp "Racial and Ethnic Differences"/ or exp Cross Cultural Differences/ or ((cultural or racial) adj3 difference*).ti,ab.<br>8. exp "Racial and Ethnic Groups"/ or ("race factor*" or "ethnic group*" or race* or racial or "racial disparit*" or "racial minorit*" or ethnicit* or "ethnic disparit*" or "ethnic minorit*" or ethnic* or minorit* or culture*).ti,ab.<br>9. exp Blacks/ or (negro* or black* or "african american*" or "afro american*" or afroamerican*).ti,ab.<br>10. exp "Latinos/Latinas"/ or ("spanish american*" or "Puerto Rican*" or latina* or Cuban* or hispanic* or latino* or "mexican american*").ti,ab.

	11. exp American Indians/ or ("native american*" or "american indian*" or indigenous*).ti,ab.exp<br>12. Asians/ or (asian* or "pacific islander*").ti,ab.<br>13. exp Alaska Natives/ or exp Native Alaskans/ or exp Inuit/ or ("american indian*" or "american native*" or "alaska* native*" or "alaska* indigenous people*" or "indigenous alaska*" or "native alaska*" or inuit* or eskimo* or inuk* or aleut* or kalaallit* or inupiat* or alaska*).mp.<br>14. 6 or 7 or 8 or 9 or 10 or 11 or 12 or 13<br>15. 4 and 5 and 14<br>16. Limit 15 to (english language and yr="2009 - 2024")<br>17.
PubMed	((("Treatment Outcome"[Mesh] OR "Patient Outcome Assessment"[Mesh] OR "Patient Reported Outcome Measures"[Mesh]) OR ("functional outcome*"[Title/Abstract] OR outcome*[Title/Abstract])) AND (("Brain Injuries, Traumatic"[Mesh]) OR ("traumatic brain injur*"[Title/Abstract] OR "tbi"[Title/Abstract]) OR ("Brain Injuries"[Mesh]) OR ("brain injur*"[Title/Abstract] OR "head injur*"[Title/Abstract]) OR ("Brain Concussion"[Mesh] OR "Post-Concussion Syndrome"[Mesh]) OR (concuss*[Title/Abstract])) AND (("Minority Health"[Mesh] OR "Minority Groups"[Mesh]) OR ("health disparit*"[Title/Abstract]) OR ("Race Factors"[Mesh] OR Ethnic Groups[Mesh]) OR ("race factor*"[Title/Abstract] or "ethnic group*"[Title/Abstract] or race*[Title/Abstract] or racial[Title/Abstract] or "racial disparit*"[Title/Abstract] or "racial minorit*"[Title/Abstract] or ethnicit*[Title/Abstract] or "ethnic disparit*"[Title/Abstract] or "ethnic minorit*"[Title/Abstract] or ethnic[Title/Abstract] or minorit*[Title/Abstract] or culture*[Title/Abstract] OR "cultural difference*"[Title/Abstract] OR "racial difference*"[Title/Abstract]) OR (African Americans[Mesh]) OR (negro*[Title/Abstract] OR black*[Title/Abstract] OR "african american*"[Title/Abstract] OR "afro american*"[Title/Abstract] OR afroamerican*[Title/Abstract]) OR (Hispanic Americans[Mesh]) OR ("spanish american*"[Title/Abstract] or "Puerto

	Rican"[Title/Abstract] or latina*[Title/Abstract] or Cuban*[Title/Abstract] or hispanic*[Title/Abstract] or latino*[Title/Abstract] or "mexican american*"[Title/Abstract]) OR (American Natives[Mesh] OR "Indians, North American"[Mesh]) OR ("native american*"[Title/Abstract] OR "american indian*"[Title/Abstract] OR indigenous*[Title/Abstract]) OR (Asian Americans[Mesh]) OR (asian*[Title/Abstract] OR "pacific islander*"[Title/Abstract]) OR ("American Indian or Alaska Native"[Mesh] OR "Alaska Natives"[Mesh] OR "Inuit"[Mesh]) OR ("american indian*"[Title/Abstract] OR "american native*"[Title/Abstract] OR "alaska* native*"[Title/Abstract] OR "alaska* indigenous people*"[Title/Abstract] OR "indigenous alaska*"[Title/Abstract] OR "native alaska*"[Title/Abstract] OR inuit*[Title/Abstract] OR eskimo*[Title/Abstract] OR inuk*[Title/Abstract] OR aleut*[Title/Abstract] OR kalaallit*[Title/Abstract] OR inupiat*[Title/Abstract] OR alaska*[Title/Abstract]))) AND ((2009:2024/1/10[pdat]) AND (english[Filter])))
